# Air pollution and mortality in the Canary Islands: a time-series analysis

**DOI:** 10.1186/1476-069X-9-8

**Published:** 2010-02-12

**Authors:** Elena López-Villarrubia, Ferran Ballester, Carmen Iñiguez, Nieves Peral

**Affiliations:** 1 Dirección General de Salud Pública. Gobierno de Canarias, Alfonso XIII, 4. 35003 Las Palmas de Gran Canaria, Spain; 2 Valencian School of Studies for Health-EVES c/Joan de Garay, 21; 46017 Valencia, Spain; 3 CIBER en epidemiología y salud pública (CIBERESP), Barcelona, Spain; 4 Center for Public Health Research (CSISP), Valencia, Spain

## Abstract

**Background:**

The island factor of the cities of Las Palmas de Gran Canaria and Santa Cruz de Tenerife, along with their proximity to Africa and their meteorology, create a particular setting that influences the air quality of these cities and provides researchers an opportunity to analyze the acute effects of air-pollutants on daily mortality.

**Methods:**

From 2000 to 2004, the relationship between daily changes in PM_10_, PM_2.5_, SO_2_, NO_2_, CO, and ozone levels and daily total mortality and mortality due to respiratory and heart diseases were assessed using Generalized Additive Poisson models controlled for potential confounders. The lag effect (up to five days) as well as the concurrent and previous day averages and distributed lag models were all estimated. Single and two pollutant models were also constructed.

**Results:**

Daily levels of PM_10_, PM_2.5_, NO_2_, and SO_2 _were found to be associated with an increase in respiratory mortality in Santa Cruz de Tenerife and with increased heart disease mortality in Las Palmas de Gran Canaria, thus indicating an association between daily ozone levels and mortality from heart diseases. The effects spread over five successive days. SO_2 _was the only air pollutant significantly related with total mortality (lag 0).

**Conclusions:**

There is a short-term association between current exposure levels to air pollution and mortality (total as well as that due specifically to heart and respiratory diseases) in both cities. Risk coefficients were higher for respiratory and cardiovascular mortality, showing a delayed effect over several days.

## Background

Since the 1990s, a number of studies have shown that daily pollution variations in urban ambient air are associated with an increase in mortality even when the fluctuations are below international standards [1,2]. The results of the EMECAS Project [3] (Spanish Multicenter Study on Air Pollution and Health), which was conducted on the basis of data from 13 Spanish cities with an overall population of over 10 million inhabitants, corroborated the existence of an association between air pollution and mortality among the urban Spanish population, indicating higher risk estimates for specific causes, mainly respiratory diseases. This study, however, did not include any locations in the Canary Islands.

The island factor of the cities of Las Palmas de Gran Canaria (L/P de Gran Canaria) and Santa Cruz de Tenerife (S/C de Tenerife), in addition to the meteorology that characterizes the Canary Islands as a whole, create a particular setting that influences the air quality in both cities. The predominance of trade winds, which blow almost constantly from May to October, facilitates the dispersion of primary pollutants in this urban environment, whereas their proximity to Africa favors the arrival of natural particulate matter to the islands [4]. The special environmental and climatic features, including mild temperatures, limited temperature fluctuation, irregular and scarce rainfall, trade winds, and seasonality of African air mass intrusions, offer a opportunity to analyze the relation between air pollutants and their short-term health effects in the two Canary capitals. The CAS Project (in Spanish, *Canarias, Atmósfera y Salud *or "The Canary Islands, Atmosphere, and Health") was launched with this aim.

In this article, we present our results concerning the short-term impact of exposure to air pollutants on total mortality and that due to respiratory and heart diseases in both cities between the years 2000 and 2004.

## Methods

**Figure 1:**
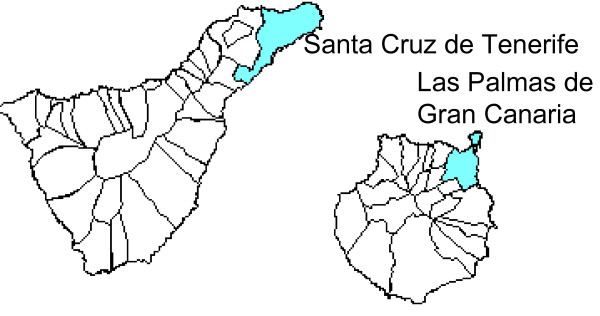
**Cities of Las Palmas de Gran Canaria and Santa Cruz de Tenerife**.

Las Palmas de Gran Canaria and Santa Cruz de Tenerife are located in the northeastern sections of the islands of Gran Canaria and Tenerife, respectively (Figure 1). Although they share many climatic features, their specific environments define certain differences between them. The city of L/P de Gran Canaria, with a population of approximately 374,000 inhabitants, is for the most part located on an isthmus. This allows free circulation of the northeast trade winds, thus providing regular dispersive conditions. Road traffic is the main anthropogenic source of pollution. In S/C de Tenerife, with a population of nearly 219,000 inhabitants, such dispersive conditions are diminished when the winds come from the east/southeast because the Anaga Massif circles the city to the north/northwest. Moreover, in addition to the city's road traffic, there is also the influence of industrial pollution from a local oil refinery.

The low troposphere over the Canary Islands is strongly stratified. There are two main types of African dust outbreaks over the islands: low altitude atmospheric intrusions (from October to April) and high altitude atmospheric intrusions (in summer). The speed/direction analysis in the air thus reflects only the influence of the trade winds with no clear patterns of transport from Africa, which is why we did not consider it to be a confounder in the statistical analysis.

## Data

We obtained daily death reports for the two cities for the years 2000 to 2004 from the Mortality Register of the Canary Islands Regional Authority. In line with the International Classification of Diseases, Tenth Revision (ICD-10; WHO 1999), we analyzed daily death counts of all-causes, excluding deaths from external causes (ICD-10: A00-R99), daily death counts of all heart diseases (ICD-10: I20-I25, I46-I50, R98-99, R09.2) and daily death counts of all respiratory diseases (ICD-10: J00-J95, J97-J99, R09.1) in all age groups.

Air pollution data were obtained from the Air Quality Network. Two monitoring sites were selected in S/C de Tenerife and one in L/P de Gran Canaria, with valid data for over 94% of the days (Table 1). Based on the hourly concentration measures representative of each city, the following variables were constructed: 24-hr averages of particulates (PM_10_-24 h and PM_2.5_-24 h), nitrogen dioxide (NO_2_-24 h) and sulfur dioxide (SO_2_-24 h), as well as the 8-hr maximum moving averages of ozone (O_3_-8 h) and carbon monoxide (CO-8 h).

**Table 1:** Descriptive statistics of daily pollutant levels, death counts, and meteorological variables from 2000 to 2004

	Las Palmas de Gran Canaria					Santa Cruz de Tenerife				
	**Mean**	**P50**	**sd**	**min**	**max**	**Mean**	**P50**	**sd**	**min**	**max**

*Air pollutant concentrations*										

▪ PM_10_-24 h (μg/m^3^)	37.7	29.2	40.6	8.7	612.4	42.3	31.7	41.7	13	622

▪ PM_25_-24 h^(a) ^(μg/m^3^)	16.4	13	15.2	2.4	210.6	15.2	11.4	14.3	3.5	227.6

▪ SO_2_-24 h (μg/m^3^)	8.1	6.9	4.2	1.5	44.4	14.1	10	12.6	2.3	145.9

▪ NO_2_-24 h (μg/m^3^)	45.8	46.4	14.8	7.2	104.7	30.3	26.9	16.4	5.4	93.3

▪ CO-8 h (μg/m^3^)	0.9	0.7	0.5	0.1	6.8	1.1	1.0	0.7	0.1	4.5

▪ O_3_-8 h (μg/m^3^)^(b)^	37.9	36.6	17.4	4.2	114.8	53.0	52.2	15.1	15.0	101.4

*Daily death counts*										

▪ Total	7.4	7.0	2.8	0.0	19.0	4.4	4.0	2.2	0.0	14.0

▪ Heart disease	1.7	2.0	1.3	0.0	9.0	0.9	1.0	1.0	0.0	6.0

▪ Respiratory	0.7	0.0	0.9	0.0	5.0	0.4	0.0	0.6	0.0	4.0

*Meteorological measures*										

▪ Mean Temperature (°C)	21.1	21	2.6	14.4	32.5	21.3	20.8	3.0	13.9	33.6

▪ Relative humidity (%)	65.5	65.8	9.4	22.3	95	62.2	61.8	7.6	31.3	85.8

▪ Barometric pressure (mbar)	1012.6	1008.7	3.45	986.3	1023.7	1014.3	1014.2	3.60	998.8	1026.6

(a) PM_2.5 _only from 2001 to 2004

(b) Entire annual period

Occasionally, during the episodes of African dust, average daily levels of PM_10 _and PM_2.5 _reached 600 and 200 μg/m^3^, respectively (Table 1). In order to avoid the influence of these extreme values on estimates of the association, and given that most of the studies analyzing the shape of the exposure-response relationship between PM and mortality have observed a linear dependence between PM_10 _and mortality below 150 μg/m^3^, days with average levels of PM_10 _over this concentration (1.9% of days in L/P de Gran Canaria and 2.4% in S/C de Tenerife) were excluded from this analysis. Because dust events also have an impact on PM_2.5 _levels [5], those days with average PM_2.5 _levels over 75 μg/m^3 ^(1% of days in L/P de Gran Canaria and 0.8% in S/C de Tenerife) were also excluded.

### Statistical analysis

The association between air pollution and mortality was investigated separately in each city using Poisson regression models and allowing for overdispersion. A smooth function of time was used to remove potential confounding effects of long term trends and seasonality. Smooth functions were also used to control for the potentially confounding effects of weather and influenza, because their relationship with the outcome is expected to be nonlinear. In particular, lags 0 to 1 and lags 2 to 4 averages of temperature, relative humidity, and barometric pressure were considered as meteorological variables. Daily counts of influenza were estimated by smoothing the series of weekly counts divided by 7. Lags 0 to 6 average of the daily estimated influenza was considered as influenza variable. Dummy variables were included to control for the effect of calendar variables, i.e.: day of the week, holidays, and unusual events, such as medical strikes. Terms for temperature, influenza, and day of the week were always present in the model. The presence of the remaining variables was tested by means of the likelihood ratio test (p ≤ 0.05).

Penalized regression splines, implemented by Wood in R [6], were used as smoothing functions with thin-plate regression splines used as basis functions [6,7]. Preliminary decisions on the number of bases were in line with APHEA2 methodology [8]. The number of basis functions was chosen to be 10 per year (i.e. 50 total) for time and 10 for the remaining non-linear terms. The smoothing parameter for time was chosen by minimizing the absolute value of the sum of partial autocorrelation (PAC) of the residuals; however, a minimum of 1 degree of freedom per year was required. Following this procedure, in L/P de Gran Canaria the effective *df*s for the time trend for total mortality and mortality from heart and respiratory diseases were 9, 8, and 12, respectively, while 5 resulted for all three mortality outcomes in S/C de Tenerife. The smoothing parameter for the remaining smoothers was chosen automatically by means of generalized cross-validation [6].

For each pollutant, mortality outcome, and city, the exposure on the current day (lag 0) was introduced into the basal model as a smoothing term. We assumed linearity when the effective *df *values were close to 1. Exposure on the current day and lags up to 5 days were also examined. Given that a 2-day moving average of air pollutants has been described as fitting better than any single day's results [9,10], a model with concurrent and previous day average (lag01) was fitted. To account for serial correlation in the residuals, we added autoregressive terms into the model as appropriate [11]. The magnitude of the association was expressed as the percentage of change in death risk for each 10 μg/m^3 ^increase (1 mg/m^3 ^for CO) in each one of the pollutant indicators and CI 95%.

**Figure 2:**
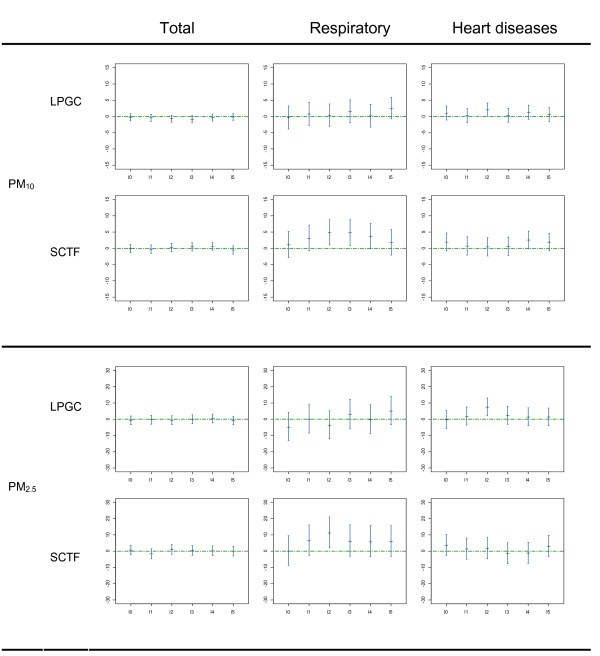
**Percent increase for each 10 μg/m^3 ^increment of PM_10 _and PM_2.5 _Percent increase in daily mortality categories and CI 95% per 10 μg/m^3 ^increment of PM_10 _and PM_2.5 _by city from 2000 to 2004**. *LPGC: Las Palmas de Gran Canaria city; SCTF: Santa Cruz de Tenerife city. X axis: each of the analyzed lags. Y axis: percent of mortality risk increase*.

**Figure 3:**
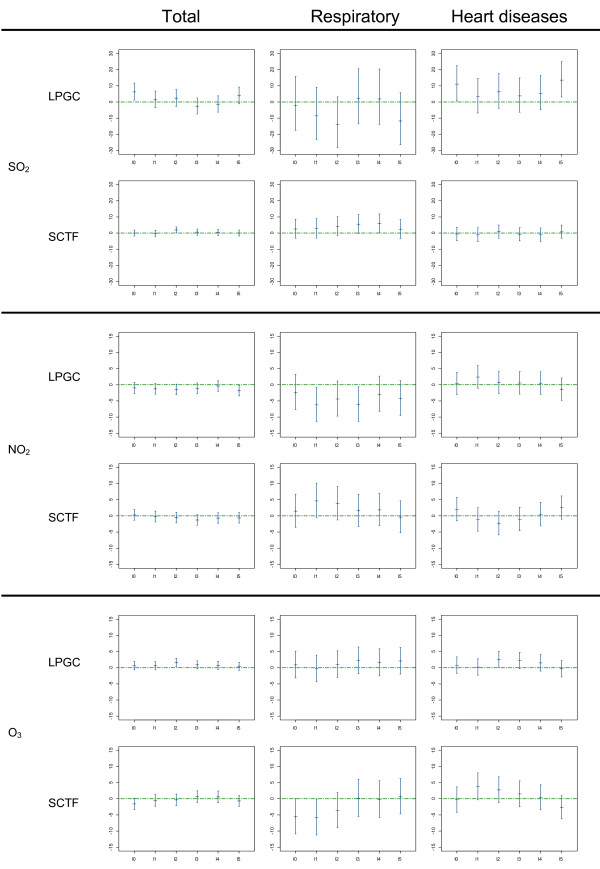
**Percent increase for 10 μg/m^3 ^increment of SO_2_, NO_2_, and O_3 _Percent increase in daily mortality categories and CI 95% per 10 μg/m^3 ^increment of SO_2_, NO_2_, and O_3 _by city from 2000 to 2004**. *LPGC: Las Palmas de Gran Canaria city; SCTF: Santa Cruz de Tenerife city. X axis: each of the analyzed lags. Y axis: percent of mortality risk increase*.

We checked the linearity of the relation between air pollution and mortality, assuming it to be the case if the effective *df *values estimated by the GCV function of the model were close to 1 when introducing the pollutants as smoothing functions of air-pollution indicators. The linear model provided the best results in 68.1% of the cases (147 out of 216 models). To provide a better interpretation and comparability of data as well as to allow for a closer examination of their lag structure, the results from linear models are presented in figures 2 and 3.

Due to O_3 _transboundary transport, the highest levels of ozone are usually observed from February to May in L/P de Gran Canaria and between March and May in S/C de Tenerife [12,13]. Consequently we examined the effect of ozone for the entire annual period and as well as for the period restricted to the aforementioned months in each city (basically during springtime).

To assess whether there was confounding by other pollutants in the estimates, two-pollutant models were calculated, simultaneously including all the possible pairs of pollutants and subsequently examining the variations in the coefficients of the one pollutant models after each of the other pollutants had been included.

To investigate the cumulative associations between air pollutants and daily mortality counts up to 5 days after exposure, unconstrained distributed lag models were used [14,15]. From these we obtained cumulative estimates as the sum of the estimated coefficients for any given lag (lags 0 to 5).

Finally, in order to examine differences in this association among different age groups, we analyzed the effects of pollution on total mortality in people above and below the age of 70.

## Results

Table 1 summarizes the distribution of daily levels of air-pollutants, meteorological variables, and mortality count data for this period in the two cities. During the study period, the median daily concentrations of SO_2 _and NO_2 _were 6.9 and 46.6 μg/m^3^, respectively, in L/P de Gran Canaria, and 10 and 26.9 μg/m^3^, respectively, in S/C de Tenerife. The medians of the 8 hr maximum moving average of CO and O_3 _were 0.7 mg/m^3 ^and 36.6 μg/m^3 ^respectively in L/P de Gran Canaria, and 1.0 mg/m^3 ^and 52.2 μg/m^3 ^respectively in S/C de Tenerife.

PM_10 _was very strongly correlated with PM_2.5 _in both cities (correlation coefficient, *r *= 0.93) (Table 2) [13] while PM_2.5 _was moderately associated with SO_2_, NO_2_, and CO in S/C de Tenerife (*r *= 0.27, 0.26, and 0.31, respectively). The correlations observed were weaker in L/P de Gran Canaria (*r *= 0.07 for SO_2 _and NO_2 _and *r *= -0.08 for CO-8 h) (Table 2). O_3_-8 h (entire annual period) was negatively and moderately correlated with particulate matter and gases in S/C de Tenerife (-0.39<*r*<-0.16), but exhibited a lower correlation in L/P de Gran Canaria (*r *= -0.12<*r*<0.15).

**Table 2:** Correlation coefficients between daily air-pollutant concentrations and weather conditions in Las Palmas de Gran Canaria and Santa Cruz de Tenerife

		SO_2_- 24 h	NO_2_- 24 h	PM_10_- 24 h	PM_25_- 24 h	O_3_-8 h	CO-8 h	Temperature	Humidity	Barometric pressure
Las Palmas de Gran Canaria	SO_2_-24 h	1	0.08**	0.22**	0.07*	-0.12**	0.09**	-0.09**	-0.04	0.01
	
	NO_2_-24 h		1	0.11**	0.07**	0.15**	0.09**	-0.27**	-0.29**	0.12**
	
	PM_10_-24 h			1	0.93**	0.08**	-0.03	-0.01	-0.21**	0.03
	
	PM_25_-24 h				1	0.10**	-0.08**	-0.01	-0.21**	0.00
	
	O_3_-8 h					1	-0.06*	-0.37**	-0.15**	0.14**
	
	CO-8 h						1	0.05*	0.08**	-0.09**
	
	Temperature							1	-0.20**	-0.30**
	
	Humidity								1	0.00
	
	Barometric pressure									1


Santa Cruz de Tenerife	SO_2_-24 h	1	0.36**	0.22**	0.27**	-0.20**	0.19**	0.14**	0.06	-0.20**
	
	NO_2_-24 h		1	0.25**	0.26**	-0.39**	0.15**	-0.13**	0.10**	0.09**
	
	PM_10_-24 h			1	0.93**	-0.18**	0.18**	0.04	-0.06*	0.02
	
	PM_25_-24 h				1	-0.16**	0.31**	0.06*	-0.04	-0.02
	
	O_3_-8 h					1	-0.14**	-0.22**	-0.35**	0.06**
	
	CO-8 h						1	0.20**	-0.02	-0.08**
	
	Temperature							1	-0.19**	-0.26**
	
	Humidity								1	-0.12**
	
	Barometric pressure									1

*p < 0.05

**p < 0.01

Figures 2 and 3 present the estimated effects of air-pollutants at each lag along with the CI 95% for total and cause-specific mortality (except for CO, which did not show any significant result). 58.3% of the estimated coefficients were positive. In these cases, the effect of a single day's exposure to the analyzed pollutants was manifested across several days. The magnitude of the association was higher for cause-specific mortality, particularly that related to respiratory diseases in S/C de Tenerife, which showed a lag effect on mortality risk.

We found that the two differently sized fractions of particulate matter were both positively associated with respiratory and heart disease mortality in the two cities (Figure 2). The effects of PM_2.5 _on mortality from heart disease in L/P de Gran Canaria and on respiratory mortality in S/C de Tenerife were statistically significant in lag 2. Moreover, in S/C de Tenerife, for each increase of 10 μg/m^3 ^in average daily PM_10_levels, respiratory mortality increased by 4.9% (CI 95%: 1.1 to 8.8) at a lag of 2 days. The response patterns for the analyzed lags were quite similar for PM_10 _and PM_2.5 _in each city, although the PM_2.5_coefficients observed were higher.

Daily SO_2 _levels were significantly associated with total mortality risk on the same day of exposure in L/P de Gran Canaria and at lag 2 in S/C de Tenerife, where a risk increase of 2.0% was observed (CI 95%: 0.17 to 3.84). Nevertheless, the highest impact was observed for mortality due to heart disease in L/P de Gran Canaria (lag 5) (Figure 3) and on respiratory mortality in S/C de Tenerife (lag 4), with an increase of 5.9% (CI 95%: 0.2 to.11.8).

When we analyzed the data for the entire annual period, both cities showed a similar ozone pattern in relation to death caused by heart disease, with a lag effect of between 1 and 4 days, although this association was only statistically significant in L/P de Gran Canaria (lag 2) with an increase of 2.5% (CI 95%: 0.03 to 5.1). In this city O_3 _was also significantly associated with total mortality in lag 2. In S/C de Tenerife we found a statistically negative association between O_3 _and respiratory diseases at lag 1 (Figure 3), which disappeared when we analyzed the series restricted to the spring period. In this restricted analysis, the estimated coefficients were basically unchanged for both cities. In L/P de Gran Canaria, increases in O_3 _levels were also significantly associated with mortality from heart disease (lags 2 and 3) as well as with total mortality (lag 3). In S/C de Tenerife, lag 5 was the most significant period in association with mortality from heart disease, with an increase of 1.44% (CI 95%: -6.6 to 4). This was the main difference, since the association with total and respiratory mortality remained the same.

In S/C de Tenerife we observed a 4.6% increase (CI 95%: -0.5 to 10) in respiratory mortality at lag 1, which was associated with an increase of 10 μg/m^3 ^in NO_2 _levels, declining to 0 at lag 5 (Figure 3). In L/P de Gran Canaria, the risk estimates for lags 1 and 3 were negative and statistically significant, and persisted in the two-pollutant model.

With regard to associations between CO and the analyzed mortality outcomes, although the estimated coefficients were mainly positive, we found no significant results.

Overall, the association of particulate matter and gases with mortality was not confounded. The only exceptions were the aforementioned association between PM_2.5 _and respiratory mortality (confounded by SO_2_) and the negative association between ozone (throughout the entire study period) and respiratory mortality, which disappeared after introducing NO_2 _levels into the model (both in S/C de Tenerife). Regarding the results among different age groups, we observed no remarkable differences in total mortality estimates for people 70 years old and older as compared with mortality for people under 70.

Tables 3 and 4 show the percentage of change in mortality risk for the analyzed causes of death related to a 10 μg/m^3 ^increase in the exposure variables using the average of the concurrent and previous day (lag01) and the unconstrained distributed lag model (DLM). PM_10 _estimates showed greater overall effects in DLM than in models using only single-day exposure (lag 0) or lag01 estimates. DLM for PM_2.5 _estimates showed greater overall effects than lag 0 or lag01 in S/C de Tenerife for both respiratory and heart disease mortality (table 4) while in L/P de Gran Canaria this was only the case for mortality due to heart disease (table 3). A similar pattern was found for SO_2 _and NO_2 _estimates while for the other pollution indicators and causes, no systematic pattern was observed. In the DLM, only daily SO_2 _levels were positive and significantly associated with mortality from heart diseases; in L/P de Gran Canaria, for example, an 18.5% increase was found (CI 95%: 2.1 to 37.4) while in S/C de Tenerife, the association was with respiratory mortality, with an increase of 12.5% (CI 95%: 1.07 to 25.2). Positive estimates were also found for other causes and pollutants, but for respiratory mortality in S/C de Tenerife, most of the risk estimates were positive with the DLM and lag01 models while the same held true in L/P de Gran Canaria for mortality from heart disease. Overall, this corroborates the results from the single-day estimates (Figures 2 and 3).

**Table 3:** Percent change in daily mortality categories and CI 95% for each 10 μg/m^3 ^increase (1 mg/m^3 ^for CO). Las Palmas de Gran Canaria

		Total		Respiratory		Heart diseases	
	
Pollutant		%	CI 95%	%	CI 95%	%	CI 95%
**PM_10_-24 h**	Lag01^a^	-0.57	-1.90, 0.77	-0.27	-4.71, 4.37	1.42	-1.20, 4.11
	
	DLM(05)^b^	-1.39	-3.43, 0.69	0.24	-6.75, 7.74	3.61	-0.49, 7.87

**PM_2.5_-24 h^c^**	Lag01	-0.91	-4.00, 2.28	-4.22	-13.80, 6.40	1.93	-4.23, 8.49
	
	DLM(05)	-2.60	-6.99, 1.99	-8.63	-21.34, 6.13	2.45	-6.11, 11.80

**SO_2_-24 h**	Lag01	5.23	-0.86, 11.70	-6.83	-24.25, 14.6	9.22	-3.08, 23.08
	
	DLM(05)	4.23	-3.45, 12.61	-12.5	-32.28, 14.27	18.46	2.11, 37.43

**NO_2_-24 h**	Lag01	-1.36	-3.17, 0.48	-4.82	-10.52, 1.23	1.31	-2.38, 5.13
	
	DLM(05)	-2.17	-4.31, 0.02	-7.08	-13.70, 0.04	0.35	-4.12, 5.02

**CO-8 h**	Lag01	-2.26	-6.99, 2.71	8.95	-8.30, 29.41	-7.30	-16.51, 2,93
	
	DLM(05)	-2.56	-8.74, 4.05	11.20	-11.85, 40.29	-4.05	-16.26, 9.94

**O_3_-8 h^d^**	Lag01	0.54	-1.58, 2.71	0.51	-5.93, 7.4	0.35	-3.90, 4.79
	
	DLM(05)	1.54	-1.09, 4.23	1.91	-5.89, 10.36	3.25	-2.09, 8.89

^a^Lag01: Average of levels of air pollutant during concurrent day (lag 0) and previous day (lag 1).

^b^DLM(05): Distributed lag model for lags 0 to 5.

^c^PM_2.5 _only from 2001 to 2004

^d ^Ozone restricted to spring period: Las Palmas de Gran Canaria: from February to May, Santa Cruz de Tenerife: from March to May.

**Table 4:** Percent change in daily mortality categories and CI 95% for each 10 μg/m^3 ^increase (1 mg/m^3 ^for CO). Santa Cruz de Tenerife

		Total		Respiratory		Heart diseases	
	
Pollutant		%	CI 95%	%	CI 95%	%	CI 95%
**PM_10_-24 h^a^**	Lag01^a^	0.00	-1.53, 1.55	3.18	-1.55, 8.14	1.56	-1.77, 5.00
	
	DLM(05)^b^	0.15	-2.09, 2.45	4.94	-2.59, 13.06	4.08	-0.91, 9.32

**PM_2.5_-24 h^c^**	Lag01	-0.68	-3.92, 2.67	2.30	-7.68, 13.36	3.24	-3.81, 10.80
	
	DLM(05)	0.69	-3.67, 5.25	7.4	-7.35, 24.49	3.46	-6.18, 14.10

**SO**_2_**-24 h**	Lag01	-0.11	-2.34, 2.18	3.85	-3.11, 11.30	-1.22	-6.13, 3.94
	
	DLM(05)	1.44	-1.88, 4.87	12.47	1.07, 25.16	-0.88	-8.04, 6.85

**NO**_2_**-24 h**	Lag01	0.03	-1.76, 1.85	3.41	-2.13, 9.26	0.37	-3.49, 4.38
	
	DLM(05)	-0.90	-3.09, 1.34	2.36	-4.57, 9.79	0.60	-4.23, 5.68

**CO-8 h**	Lag01	1.53	-3.25, 6.54	11.87	-3.69, 29.94	-2.35	-12.24, 8.65
	
	DLM(05)	0.03	-5.68, 6.08	-3.28	-20.96, 18.37	-0.43	-12.66, 13.50

**O_3_-8 h^d^**	Lag01	-1.88	-4.64, 0.94	-4.91	-13.14, 4.09	0.03	-6.02, 6.45
	
	DLM(05)	-1.56	-4.82, 1.82	-5.20	-15.18, 5.95	-0.84	-8.02, 6.90

^a^Lag01: Average of levels of air pollutant during concurrent day (lag 0) and previous day (lag 1).

^b^DLM(05): Distributed lag model for lags 0 to 5.

^c^PM_2.5 _only from 2001 to 2004

^d ^Ozone restricted to spring period: Las Palmas de Gran Canaria: from February to May, Santa Cruz de Tenerife: from March to May

## Discussion

The results of this study suggest the existence of a short-term relation spreading over successive days between air pollution and mortality in these two cities between 2000 and 2004. Overall, the risk estimates present a higher magnitude when associated with specific mortality outcomes, including those due to heart disease and especially with those caused by respiratory diseases. This is in good agreement with the psychopathological mechanisms described for these events [16-19].

The association between particulate matter and mortality has been studied extensively in European, North American, and Asian cities [20-22]. However, the peculiarities of the Canary Archipelago have a significant effect on the origin and composition of the particulate matter to which its citizens are exposed [5,23,24]. As previously explained in the Methods section above, extreme values for particulates were taken out of our calculations to avoid an undue influence on the risk estimates. Nevertheless, we observed a positive association between the two fractions of particulate matter and mortality for all the specific mortality outcomes analyzed (Figure 2). This association was highest for respiratory mortality in S/C de Tenerife, with positive coefficients for the various lags analyzed and with the most significant estimate at lag 2 for the two fractions. The relationship between PM_2.5 _levels and respiratory mortality was slightly confounded by SO_2 _levels, indicating a possible role of industrial sources of pollution. In L/P de Gran Canaria, we observed a similar pattern for mortality from heart disease, noting that the relationship with the PM fractions remained stable in the presence of other pollutants. The unconstrained distributed lag model showed a greater total effect than the average lag01 and concurrent day (lag 0) models, especially in PM estimates. These results are all in good agreement with diverse findings from time series analysis studies [25-29], as well as with the physiopathological mechanisms implicated in these processes [16,30,31].

SO_2 _and NO_2 _levels were moderately correlated with PM_2.5 _levels in S/C de Tenerife, but not in L/P de Gran Canaria, where more regular dispersive conditions exist. The former city additionally suffers the influence of an industrial source of pollution. On the whole, all the gases studied are consistently associated with respiratory mortality in S/C de Tenerife and with heart disease mortality in L/P de Gran Canaria, mimicking the same pattern that we observed for the association between particulate matter and mortality. Moreover, these positive associations also showed greater overall effects in the unconstrained distributed lag model. While these findings may reflect the existence of different susceptible population groups in the two cities, it is more likely that the cities have different PM compositions due to the different pollution sources found in each one.

In L/P de Gran Canaria the estimated coefficients for SO_2 _showed a considerable magnitude and wide confidence intervals (Table 3) as compared with those found for S/C de Tenerife. Because SO_2 _levels in the former city are low (Table 1), it is possible that the coefficient estimates (assuming that log RR is a linear function of SO_2 _concentration) reflect some higher and infrequent SO_2 _levels in L/P de Gran Canaria; in fact, we obtained the best model adjustment for the association with heart disease mortality, introducing its smoothing function, with a high slope below 20 μg/m^3 ^of SO_2_. In contrast, we obtained a linear association in S/C de Tenerife, which has a lower daily average of deaths, and more precise confidence intervals in respiratory mortality estimates. If we compare the results of both cities with different European meta-analyses, these estimates are consistent with the direction of the association [32,33] and closer to the Italian meta-analysis in terms of magnitude [34]. Zmirou [35] and Stieb [36] also reported the existence of consistent associations between SO_2 _levels and cardiovascular and respiratory mortality while in the APHEA 1 study [9], this association was independent of particulates, which matches our findings.

Recent time series studies have bolstered the evidence that there is a short-term association between O_3 _and mortality [37-39]. We found evidence that an increase of 10 μg/m^3 ^in O_3 _is positively associated with an increase in the mortality risk from heart disease in both cities and with an increased risk of total mortality in L/P de Gran Canaria. This is in good agreement with other studies that have described the short-term effects of O_3 _levels on heart attack risk [40]. In fact, the analysis restricted to the springtime in L/P de Gran Canaria showed a slightly more delayed effect (3 days) for the three mortality outcomes; this effect was clearly higher only for the association with heart disease mortality in this city, which in turn corresponded to the increase in total mortality. Moreover, these effects are not due to an interaction with high temperatures since the average temperature is higher during the summer. In S/C de Tenerife we observed a weaker association between O_3 _and the analyzed causes of death during spring. We confirmed these results using average concurrent and previous day (lag 01) data, as well as under the DLM. Studies reporting the association between O_3 _and mortality have found no evidence of a confounding effect due to other pollutants [36,37,41-43], nor have recent multicenter studies conducted by Bell [44] assessing the potential confounding effects of PM. All of this is in good agreement with our results.

The impact of CO on cardiovascular mortality has been described extensively, as have the mechanisms involved in that effect [45,46]. However, no significant association was found between levels of this pollutant and the analyzed causes of death in either city, although estimates were mainly positive and of higher magnitude on death caused by respiratory diseases.

It is generally accepted that one source of uncertainty in time series analysis is the exposure measurement error that potentially leads to the loss of statistical power and the underestimation of the association [47,48]. We must point out that due to the mild climate of the Canary archipelago, and particularly in these two seaside cities, ventilation inside the home is natural, with hardly any use of air-conditioning systems. Moreover, residents spend a considerable part of the day outdoors all year round. The measurements carried out by the Air Quality Network are thus quite close to the citizens' average exposure. Furthermore, observed and unobserved confounding factors can be controlled for by the use of Poisson regression models with smooth functions for confounding variables.

Notwithstanding, our study is not exempt from certain limitations. One of these is "multiple testing." We have assessed the association between 6 pollutants and 3 causes of death with 6 lags in two cities and have thus carried out 216 comparison tests. The second drawback is the lower statistical power and the resulting loss of estimate precision due to the fact that we are analyzing two independent, medium-sized cities with limited daily events, especially with regard to specific mortality outcomes. For these two reasons, it is important to balance the consistency and coherence of our results against those from other studies. Our findings are mainly in line with existing epidemiological evidence, although the magnitude of the risk estimates obtained and their precision may be less solid than the precision of multicenter studies conducted in larger cities.

Our goal, however, was to provide a global picture of the impact of air pollution on the health of the citizens of the Canary Islands, whose exposure to particulate matter is highly influenced by mineral dust. Certainly this type of analysis is common in continental urban settings, which have different air pollution patterns. This analysis thus adds an element of breadth to the body of knowledge concerning this public health issue. In the next phase of our research, we will examine concrete aspects of this first approximation, specifically analyzing PM characterizations and the influence of African air mass intrusions.

## Conclusions

Our findings indicate the existence of a short-term association between current exposure levels to air pollutants and total mortality as well as mortality due to heart and respiratory diseases in both Canary cities studied along with evidence that the association on a given day may spread over several successive days. Both indicators of particulate air pollution, PM_10 _and PM_2.5_, showed an association with heart and respiratory mortality. SO_2 _was the only pollutant associated with total mortality.

## Abbreviations

PM: particulate matter; PM_2.5_: concentration of airborne particles with aerodynamic diameter <2.5 μm; PM_10_: concentration of airborne particles with aerodynamic diameter <10 μm; GCV: generalized cross validation; DLM: distributed lag model; SO_2_: sulphur dioxide; NO_2_: nitrogen dioxide; O_3_: ozone; CO: carbon monoxide; *df*: degrees of freedom; ICD-10: International Classification of Diseases, Tenth Revision.

## Competing interests

The authors declare that they have no competing interests

## Authors' contributions

ELV: participated in the design of the study, prepared the datasets, and drafted the manuscript; FB: participated in the design of the study and helped write the manuscript; CI: participated in the design of the study, performed part of the statistical analysis, and helped write the manuscript; NP: prepared the data sets and performed the statistical analysis.

All authors have read and approved the final manuscript.
